# Direct Bonding Method for Completely Cured Polyimide by Surface Activation and Wetting

**DOI:** 10.3390/ma15072529

**Published:** 2022-03-30

**Authors:** Ying Meng, Runhua Gao, Xinhua Wang, Sen Huang, Ke Wei, Dahai Wang, Fengwen Mu, Xinyu Liu

**Affiliations:** 1 High-Frequency High-Voltage Device and Integrated Circuits R&D Center, Institute of Microelectronics, Chinese Academy of Sciences, Beijing 100029, China; mengying@ime.ac.cn (Y.M.); wangxinhua@ime.ac.cn (X.W.); huangsen@ime.ac.cn (S.H.); weike@ime.ac.cn (K.W.); wangdahai@ime.ac.cn (D.W.); xyliu@ime.ac.cn (X.L.); 2 School of Integrated Circuits, University of Chinese Academy of Sciences, Beijing 100049, China; 3 Key Laboratory of Microelectronic Devices & Integrated Technology, Institute of Microelectronics, Chinese Academy of Sciences, Beijing 100029, China; 4 SABers Co., Ltd., Tianjing 300450, China

**Keywords:** polyimide bonding, plasma activation, hydrophilic, hybrid bonding, 3D integration

## Abstract

Polymer adhesives have emerged as a promising dielectric passivation layer in hybrid bonding for 3D integration, but they raise misalignment problems during curing. In this work, the synergistic effect of oxygen plasma surface activation and wetting is utilized to achieve bonding between completed cured polyimides. The optimized process achieves a void-less bonding with a maximum shear strength of 35.3 MPa at a low temperature of 250 °C in merely 2 min, significantly shortening the bonding period and decreasing thermal stress. It is found that the plasma activation generates hydrophilic groups on the polyimide surface, and the wetting process further introduces more −OH groups and water molecules on the activated polyimide surface. The synergistic process of plasma activation and wetting facilitates the bridging of polyimide interfaces to achieve bonding, providing an alternative path for adhesive bonding in 3D integration.

## 1. Introduction

The number of transistors on electronics was supposed to increase exponentially according to Moore’s Law; however, this trend has become difficult to sustain in traditional two-dimensional integration due to the physical limits of transistor size, the high costs of processes, and the dependence on high-precision lithography equipment. Three-dimensional (3D) integration technology [1,2,3,4] has offered an alternate path to continue Moore’s law. Through this technology, the chips or wafers with different functions are separately manufactured and vertically stacked through a hybrid bonding process, hence the significance of the hybrid bonding technology. Silicon dioxide (SiO_2_) is usually used to fill gaps between metal interconnections in traditional hybrid bonding, to prevent the metal from oxidizing during the bonding process and increasing the bonding area effectively. However, the SiO_2_ surface requires high flatness and surface cleanliness to avoid electrical interconnection failure because of its higher hardness and poor deformation characteristics. Moreover, the mismatch of the coefficient of thermal expansion (CTE) between SiO_2_ and the metal may generate residual stress during the bonding process and cause reliability issues. The adhesive possesses the advantages of flexibility, higher surface roughness, and flatness tolerance that can be used to replace oxides as a buffer layer to release residual stress from bonding pressure. Therefore, adhesive/metal hybrid bonding is a promising solution for 3D integration.

**Figure 1:**
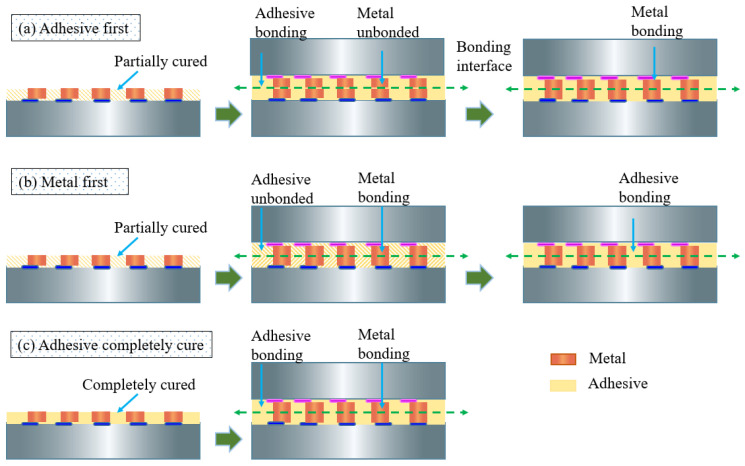
Schematic of hybrid bonding in 3D integration: (**a**) the adhesive first method, (**b**) the metal first method, and (**c**) the method in this study.

Typical hybrid bonding consists of metal/metal and adhesive/adhesive bonding; the former achieves electrical interconnection and the latter provides passivation protection and mechanical support. Most researchers have achieved the hybrid bonding process by two steps, where they separately bond metals and adhesives [5,6,7,8], as shown in Figure 1a,b. The “adhesive first” method brings benefits of being void-free and having reliable adhesive bonding but raises serious misalignment of the metal interconnection due to the fluidity and volume shrinkage of adhesives during the curing process. The “metal first” method avoids the misalignment problem, while the post-bonding curing process for adhesives causes outgassing and degrades bonding reliability. Both “adhesive first” or “metal first” methods have to involve a curing procedure in the bonding process, not only raising various problems but also increasing the bonding period. Many attempts have been made to cure the adhesive completely before hybrid bonding [9,10], as shown in Figure 1c. A high-quality dielectric passivation layer was achieved because the solvent and volatile substances of the adhesive can be removed completely. Among them, polyimide is a promising adhesive due to its advances in flexibility, chemical inertness, mechanical toughness, and thermal stability at high temperatures [11,12], which makes it withstand manufacturing processes such as metal deposition, photolithography, and wet etching processes. However, completely cured polyimides, due to their higher glass transition temperature [13], require higher bonding temperatures that are unsuitable for advanced application devices such as dynamic random-access memory (DRAM) [14] and light-emitting diodes (LEDs) [15]. Therefore, it is necessary to develop a rapid and low-temperature (<250 °C) bonding method for completed cured polyimides to achieve bonding with high throughput and reliability.

Considerable research has been conducted on the adhesion between cured polyimides. Mekaru [16] bonded two pieces of polyimide films by the thermos-compression method with a high temperature approaching the glass transition temperature; however, the possible side-effects of the high-temperature process still need to be discussed. Chen et al. [17] bonded two pieces of polyimide films by heating at 250 °C for 60 min in an oven, finding that voids inevitably existed either at the bonding interface or within the bulk films. A modified surface activated bonding (mSAB) method was proposed to bond polyimides by a thin intermediate activated metallic layer, which is promising, although the metallic layer may cause short circuit and failure when through-silicon-via technology is involved [18]. So far, it is still challenging to develop a low-temperature bonding method for ordinary polyimide to meet the requirements of low thermal budget, high throughput, and reliability. In the case of traditional wafer bonding, surface elastic deformation, hydrogen bond of −OH groups [19], and H_2_O molecular bridging [20] all play important roles in the wafer bonding. Additionally, previous studies have proven the synergistic effects of irradiation and ethanol on the bonding between polyimide and metal [21,22], demonstrating that the hydrophilic group is the key to the bonding process. Inspired by the above reports, in this study, a novel bonding method utilizing the synergistic effects of oxygen plasma surface activation and the wetting process is proposed. The polyimide surface is activated by oxygen plasma first, and the vertical bonding is achieved by thermos-compression in a nitrogen atmosphere. This method is promising in shortening the bonding process and elevating the throughput and efficiency. In this study, the oxygen plasma activation and H_2_O molecular bridge are introduced into the bonding process.

## 2. Materials and Methods

**Figure 2:**
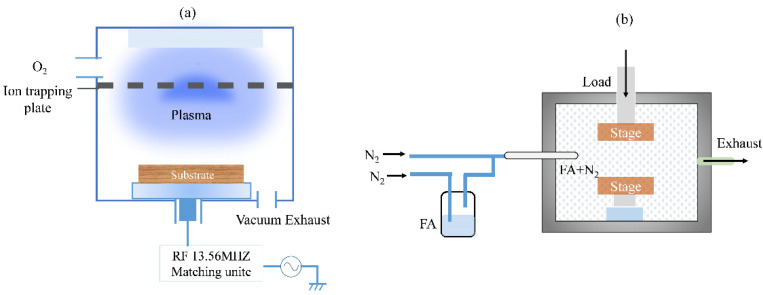
Schematic diagram of the process: (**a**) the plasma activation and the RIE equipment; (**b**) the bonding process under formic acid atmosphere. FA: formic acid.

The polyimide used in this study was synthesized from pyromellitic dianhydride and 4.4′-diaminodiphenyl ether (PMDA/4,4′-ODA), which was processed into films of 50 μm by an advanced salivating biaxial stretching and was cut to 12 mm × 12 mm. The polyimide films were sequentially sonicated with acetone, alcohol, and deionized water for 5 min to remove pollution, and were then dried with nitrogen and heated by a hotplate at 100 °C for 10 min to remove the remaining moisture. The film surfaces were activated by reactive ion etching equipment (RIE; 100M, Tailong Electronics Co., Ltd., Beijing, China) using the oxygen plasma with a power of 150 W, voltage of ~510 V, and oxygen flow of 50 sccm. The RIE chamber was first vacuumed to below 1 Pa. When the plasma was generated, oxygen with a purity of 99.99% was introduced into the chamber to maintain the pressure at ~6 Pa. This activation aimed to remove organic pollution and modify the surface to improve its hydrophilicity. Two pieces of activated polyimide films were stacked face to face with deionized water dropped at their interface, and then they were transformed into a thermos-compression chamber to achieve bonding in nitrogen under a pressure of 30 MPa. The stacked polyimide was heated from the bottom side, and pressure was applied on the top side by an aluminum block with an area of 6 mm × 6 mm to precisely control the effective bonding area. A schematic diagram of the plasma activation and bonding process is illustrated in Figure 2.

The surface morphology of the polyimide films before and after surface activation was characterized by an atomic force microscope (AFM; XE15, Park systems Co., Ltd., Suwon, Korea). The surface roughness was evaluated by the root-mean-square average (*R_rms_*) and average roughness (*R_a_*). As the irradiation of plasma is reported to cause higher roughness, compensation through the elastic deformation of the near-surface area was considered for the interface gap caused by surface roughness [23,24]. The elastic energy must be smaller than the work of adhesion (namely, the interface energy gained by the bond formation) to achieve intimate contact, expressed as Formula (1) [25,26]:

(1) $$\frac{R_{rms}^{2}}{\lambda }\leq \frac{2\left(1-\nu^{2}\right)}{\pi E}W_{A}$$

where *λ* is the wavelength of the surface profile, *E* is Young’s modulus, *ν* is Poisson’s ratio, and *W_A_* is the work of adhesion.

In addition, the surface profile is also a crucial parameter that affects the bonding strength. In this study, the power spectral density (PSD) was statistical to reveal the periodic characteristics of wavelengths in the surface profile of samples, providing a distribution of feature wavelengths from AFM profile data. The PSD represents the functional relationship between the magnitude of the surface roughness and spatial frequency (the inverse of the wavelength of the surface profile), expressed as follows:

(2) $$S_{2}\left(f_{x},f_{y}\right)=\frac{1}{L^{2}}\left[\sum_{m=1}^{N}\sum_{n=1}^{N}Z_{mn}e^{-2\pi i\Delta L\left(f_{x}m+f_{y}n\right)}{\left(\Delta L\right)}^{2}\right]$$

where *S*_2_ represents the 2D-isotropic PSD of the surface, *L* is the length of the scanned area, N denotes the number of data points for one direction, *Z_mn_* is the height at position (*m*, *n*), and *f_x_* and *f_y_* express the spatial frequencies in the *x* and *y* directions, respectively. The surface morphology result was recorded in tapping mode by AFM and converted by Fourier transform, and then the values were carried into Formula (2) to extract the wavelength of the signal.

X-ray photoelectron spectroscopy (XPS; Thermo escalab 250Xi, Thermo Fisher Scientific Inc., Waltham, MA, USA) and Fourier infrared spectrometry (FTIR; Nicolet IS10, Thermo Fisher Scientific Inc., Waltham, MA, USA) were used to evaluate the chemical state of the polyimide surface before and after plasma irradiation. The emission angle of X-ray collection electrons was set as 40°. For the bonding strength test, the upper and lower sides of the bonded polyimides were glued with aluminum blocks using acrylic glue, and shear tests were conducted on the blocks by a shear tester (MFM 1200, TRY Precision Co., Ltd., Shenzhen, China) with a shear speed of 20 μm/s. In addition, cross-sectional samples were prepared by embedding the bonded polyimide in resin and cutting them using a focused ion beam (FIB; Helios G4, Thermo Fisher Scientific Inc., Waltham, MA, USA). The bonding interface was observed with a transmission electron microscope (TEM; Themis Z, FEI Inc., Hillsboro, OR, USA).

## 3. Results and Discussion

### 3.1. Process Optimization

**Figure 3:**
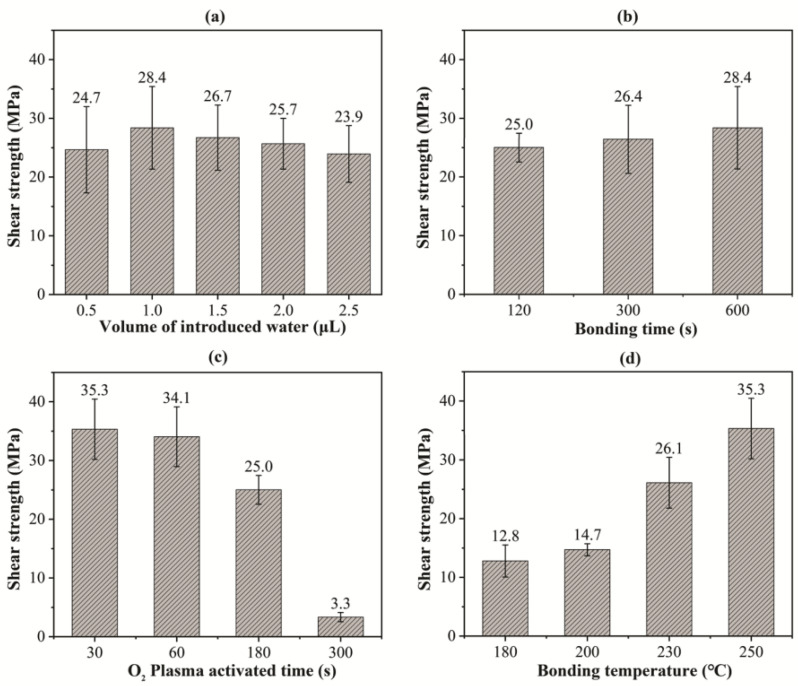
Shear strength of the polyimide bonding with different (**a**) water volumes, (**b**) bonding times, (**c**) plasma activation times, and (**d**) temperatures.

As bonding strength is a primary indicator to evaluate the reliability of vertical interconnections, the bonding process was optimized based on shear tests. The influence of volume of the introduced water, bonding time, plasma activation time, and bonding temperature on shear strength is shown in Figure 3. First, the oxygen plasma activation was set to 150 W, 180 s, and deionized water was dropped at the polyimide surfaces with different volumes (0.5–2.5 μL). The bonding temperature, the bonding time, and the pressure were set to 250 °C, 600 s, and 30 MPa, respectively. The influence of the water volume on shear strength is shown in Figure 3a. It is seen that the shear strength slightly changed with different volumes of induced water, and 1 μL of water appeared slightly superior. For the following experiments, the water volume was fixed at 1.0 μL.

The influence of bonding time on shear strength is shown in Figure 3b, where the oxygen plasma time, the bonding temperature, and the pressure were set to 180 s, 250 °C, and 30 MPa, respectively. The average shear strength was as high as 25 MPa even after bonding for merely 120 s and was slightly improved when the bonding time increased to 600 s. Although the bonding time is generally believed to positively correlate to the bonding quality, in this study, the bonding time of 120 s already obtained sufficient shear strength that met the requirements of reliability.

As the plasma activation time increased the surface roughness, it is necessary to analyze the effect of extending the plasma time on the shear strength. The bonding time and temperature were set to 120 s and 250 °C, respectively; polyimides were bonded after irradiating for 30 s, 60 s, 180 s, and 300 s with 150 W of power and 50 sccm of oxygen flow; and the results are shown in Figure 3c. The average shear strength, which met the requirement, hardly changed with the extending of plasma activation when the time was less than 60 s. When the activated time was extended to 180 s, the shear strength decreased slightly and became unstable. Especially, when the time was extended to 300 s, the shear strength greatly decreased to the extent of failure. Therefore, in this study, the plasma activation time was optimized to 30 s.

Then, the bonding time was fixed at 120 s and the oxygen plasma time was fixed at 30 s. The bonding temperature was adjusted to 180 °C, 200 °C, 230 °C, and 250 °C, and the results are shown in Figure 3d, which obviously shows the positive correlation between the bonding temperature and the shear strength. Under the bonding temperature of 180 °C, the polyimide was successfully bonded together with a shear strength of 12.8 MPa. The average shear strength reached the maximum of 35.3 MPa under the temperature of 250 °C. This correlation is reasonable because a higher bonding temperature accelerates the thermal vibration of atoms, making it easier to leave the equilibrium position and diffuse, and obtaining an interface with higher shear strength.

**Figure 4:**
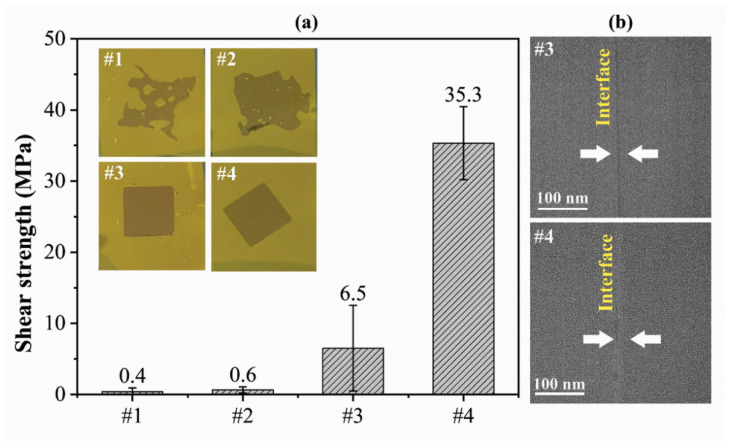
(**a**) The shear strength of specimens obtained in the comparative tests of Table 1; the inset images show the specimens after bonding under different conditions; (**b**) interface characterization of sample #3 and #4.

Additionally, the synergistic effects of plasma activation and deionized water molecules on the shear strength were proven by comparative tests, as listed in Table 1, and the corresponding shear strength is shown in Figure 4a. The inset images show the specimens after bonding under different conditions, and the well-bonded areas appeared dark. The specimens bonded without oxygen plasma activation and wetting (group #1) possessed the lowest shear strength and the smallest bonding area. This situation was not significantly improved by merely introducing water molecules on the surface (group #2), while the bonding area slightly improved. Although the plasma activation process of group #3 increased both the bonding area and the shear strength, it failed to obtain a high shear strength. The process of group #4, which introduced both water molecules and oxygen plasma activation, obtained bonding with not only a full bonding area but also a high shear strength. The bonding interfaces observed by the TEM, as shown in Figure 4b, confirmed that voids existed neither in the inner polyimides nor at the bonding interface in both cases of groups #3 and #4, while a more compact bonding with a narrower bond line was observed for group #4. The results proved the positive synergistic effects of water molecules and oxygen plasma activation, which thickened the transition zone to ~10 nm. Therefore, it was proven that the synergy of oxygen plasma activation and water molecules is essential for high-quality polyimide-polyimide bonding.

### 3.2. Effects of Oxygen Plasma Activation on the Polyimide Surface

**Figure 5:**
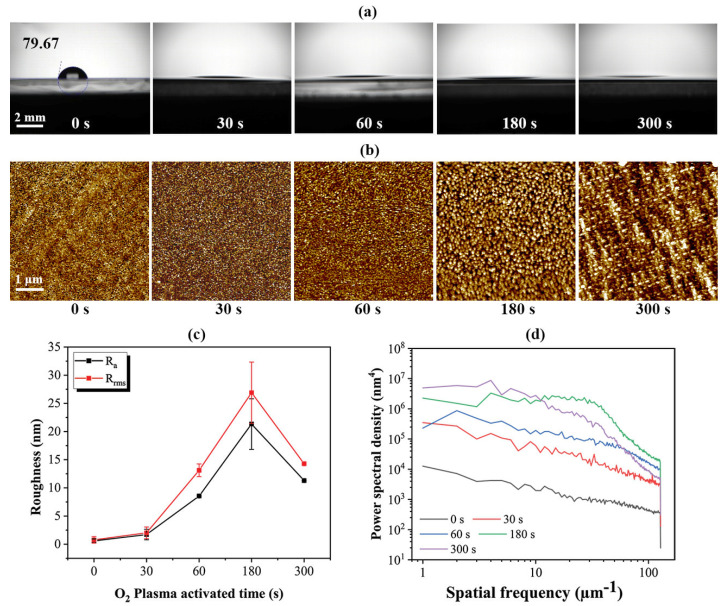
(**a**) Contact angle, (**b**) AFM results, (**c**) *R_rms_* and *R_a_* roughness, and (**d**) power spectral density of the polyimide surface after plasma activation of different periods.

The oxygen plasma activation is supposed to raise the hydrophilicity of the polyimide, and its effects were evaluated by the water-drop test. Deionized water was dropped on the polyimide surface after plasma activation of 0 s, 30 s, 60 s, 180 s, and 300 s, and the contact angles between the droplets and the surface were fit semi-automatically by capturing a picture, as shown in Figure 5a. The contact angle of polyimide before plasma activation was 79.67°, indicating the poor hydrophilicity of polyimide. The hydrophilicity was significantly improved after plasma activation of over 30 s, as the contact angle greatly decreased to below the measurement limit (10°). A further extension of activation time barely changed the contact angles.

Additionally, the surface morphology and their corresponding average surface roughness greatly changed when the activation time varied. Both *R_rms_* and *R_a_* increased dramatically with the activation time, and when the activation time exceeded 180 s, the roughness decreased instead, as shown in Figure 5c. The roughness comes from surface textures such as uneven “hillocks”; thus, it is considered that extending the plasma time caused the increase in surface roughness by raising the amplitude of hillocks on the surface, while the decrease in that at 300 s may be explained by the PSD test. The PSD results of polyimide surfaces with different activation times are shown in Figure 5d, which reveals the number of hillocks with different spatial frequencies. The PSD curves were approximately parallel and rose equally when the activation time increased from 0 to 180 s, suggesting the plasma irradiation not only increased the amplitude of hillocks but also raised the number of hillocks in all sizes. After irradiation for 180 s, the hillocks with a spatial frequency from 30 to 60 μm^−1^ increased significantly; namely, many mid-sized hillocks formed, which can also be observed in Figure 5b. When the activation time further extended to 300 s, the number of hillocks with spatial frequencies less than 10 μm^−1^ still raised significantly, while that above 10 μm^−1^ became even lower because nanosize hillocks started to agglomerate under plasma irradiation, producing many larger micro-hillocks. It is considered that the texture on the bonding surfaces greatly affected the bonding quality, because the shear strength obviously decreased when micro-hillocks formed on the surface when the activated time extended to 180 and 300 s, as shown in Figure 5c. These micro-hillocks are believed to reduce the actual contact area and create gaps that hindered atom diffusion, eventually causing debonding.

**Figure 6:**
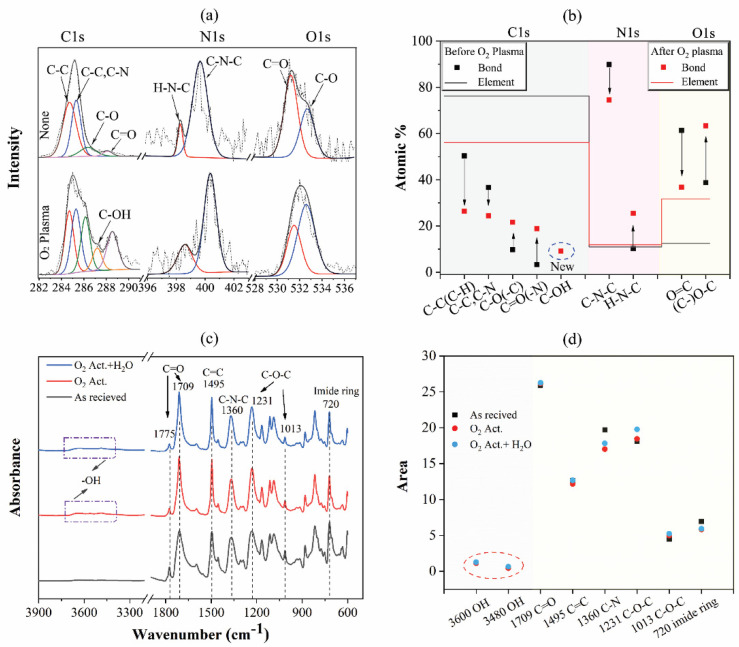
The status of polyimide surfaces before and after the plasma activation: (**a**) C 1s, N 1s, and O 1s core-level XPS spectra; (**b**) the ratio of the functional groups; (**c**) the infrared absorption spectrum obtained via FTIR tests; (**d**) the area of feature functional groups in spectra. Act.: activation.

XPS and FTIR were used to further analyze the modification of functional groups of the polyimide surface. The angular-resolved XPS with high-resolution accuracy was used to quantitatively verify the changes in the elements and functional groups before and after the plasma activation. The emission angle of X-ray collection electrons was set as 40° to detect elements and chemical bonds of the polyimide surface. The C 1s, N 1s, and O 1s spectra of polyimide before and after plasma activation are shown in Figure 6a. The C 1s spectrum was deconvoluted into four components: C−C(C−H) in ODA at 284.8 eV, C−N in ODA and C−C(C−H) in PMDA at 285.3 eV, C−O in ODA at 286.1 eV, and C=O in PMDA at 288.6 eV. The N 1s spectrum was deconvoluted into two components: the C−N−C in PMDA at 400.4 eV and C−N−H in PMDA at 398.5 eV. The O 1s spectrum was deconvoluted into two components: the O−C in ODA at 533.2 eV and the O=C in PMDA at 531.0 eV. After plasma treatment, as the rough comparison between the two spectra, the total content of C element decreased, while those of O and N elements increased obviously. For ease of understanding, the XPS results of polyimide after activation are explained by the element spectrum, addressed as follows: (I) In the C 1s spectrum, the ratio of C−C and C−C/C−N bonds decreased, while that of C−O and C=O bonds increased after the oxygen plasma activated. Moreover, a new hydrophilic group of C−OH bonds at 287 eV emerged. (II) In the N1s spectrum, the ratio of C−N−C bonds decreased, and that of C−N−H bonds increased. This reveals that oxygen plasma broke the bonds of the long-chain molecule to become short-chain molecules on the polyimide surface. (III) In the O 1s spectrum, the ratio of C−O bonds increased, and that of the C−O to C=O bonds was 1.72, which approached the ratio of (C−O + C−OH) to C=O (1.62), as shown in the C1s spectrum in Figure 6b. Therefore, the composition of C−O−C in the O 1s spectrum contained two parts: the C−O bond in ODA and the new C−OH bond.

In addition, the FTIR results of the polyimide before and after plasma activation are shown in Figure 6c,d. In both samples, the C=O bending in the imide ring at 720 cm^−1^; the C−O−C stretching and C−O−C asymmetrical stretching at 1013 cm^−1^ and 1231 cm^−1^, respectively; the C−N stretching at 1360 cm^−1^; the aromatic C=C ring stretching at 1495 cm^−1^; and the C=O asymmetrical stretching and C=O symmetrical stretching at peaks of 1709 cm^−1^ and 1775 cm^−1^, respectively, were detected. The area of C=O symmetrical stretching at 1775 cm^−1^ and the C−O−C stretching at 1231 cm^−1^ and 1013 cm^−1^ slightly increased after the plasma activation compared to functional groups as-received, while the area of the imide ring at 720 cm^−1^ and the C−N stretching 1360 cm^−1^ decreased. These results indicate that the oxygen plasma broke the bond of C−N−C and the imide ring, and reacted with the polyimide to produce new hydrophilic groups such as C=O and C−O. The results are consistent with those previously reported [27,28] where oxygen plasma will break the imide rings and phenyl groups at the surface of the polyimide film. Moreover, new OH groups were detected on the polyimide surface after plasma activation and the wetting process, as highlighted by the red circle in Figure 6d, and the surface after wetting also exhibited higher C−O−C and OH signals.

**Figure 7:**
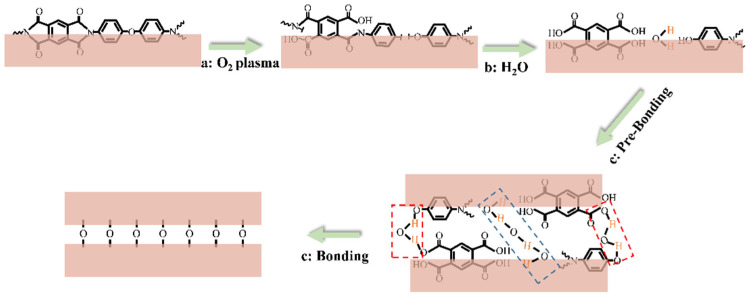
Schematic of polyimide surface activation and wetting bonding method.

In general, the XPS results show that the oxygen plasma tended to break C−C and C−N bonds, which was beneficial to the formation of C−O and C=O groups on the polyimide surface, and these results matched well with the FTIR results. A schematic of polyimide surface activation and the wetting bonding method is illustrated in Figure 7. The oxygen plasma activation introduces low-density hydrophilic groups on the polyimide surface, effectively enhancing the adsorption of water molecules, which brings considerable high-density OH groups. First, the adsorbed water molecules facilitate the generation of a pre-bonding at the bonding interfaces [29]; moreover, the considerable hydrophilic groups on the surface cause the generation of bridging bonds between polyimides, which provide a compact contact of atoms. Such compact contact promotes the generation of stable covalent bonds under appropriate bonding times and pressures, hence the improvement in bonding quality. This synergistic effect explains the results in Figure 4, that neither the plasma activation nor the hydration method is sufficient to achieve satisfied bonding alone. A reliable polyimide/polyimide bonding provides mechanical support and protection for metal interconnection, and simplifies the semiconductor processing in subsequent manufactures.

## 4. Conclusions

In summary, completely cured polyimides were directly bonded by the process involving O_2_ plasma activation and wetting procedures. By the optimized process, polyimide/polyimide bonding with a high shear strength of 35.3 MPa was achieved in 2 min at 250 °C and under a pressure of 30 MPa. The synthetic effect of oxygen plasma activation and wetting molecules was proven to promote the high-quality bonding interface between the polyimides. It was found that the oxygen plasma broke the long-chain molecules into short-chain molecules and created hydrophilic groups, with the adsorbed water introducing considerable hydrophilic groups with high-density bridging at interfaces; thus, the bonding was promoted. However, a long-time plasma activation will generate micro-hillocks that increase surface roughness, hindering bonding in return. The proposed bonding process shortened the bonding period and increased the bonding strength significantly, and it is promising for 3D integration processes. Importantly, although the proposed process was investigated for the polyimide/polyimide interface, this study revealed the possibility of scaling the mechanism to other polymer dielectric passivation layers such as PEEK and PMMA.

## Figures and Tables

**Table 1:** Comparative tests with or without plasma activation and water molecules assisting.

Group	O_2_ Plasma Activation	H_2_O
#1	×	×
#2	×	√
#3	√	×
#4	√	√

## Data Availability

Data is contained within the article.
